# Cervical auscultation in the diagnosis of oropharyngeal aspiration in children: a study protocol for a randomised controlled trial

**DOI:** 10.1186/1745-6215-14-377

**Published:** 2013-11-07

**Authors:** Thuy T Frakking, Anne B Chang, Kerry-Ann F O’Grady, Katie Walker-Smith, Kelly A Weir

**Affiliations:** 1 Queensland Children’s Medical Research Institute, The University of Queensland, Brisbane, Australia; 2 Speech Pathology Department, Royal Children’s Hospital, Brisbane, Australia; 3 Queensland Children’s Respiratory Centre, Royal Children’s Hospital, Brisbane, Australia; 4 Child Health Division, Menzies School of Health Research, Charles Darwin University, Darwin, Australia; 5 Queensland Children’s Medical Research Institute, Queensland University of Technology, Brisbane, Australia; 6 Queensland Children’s Medical Research Institute, Level 4 Foundation Building, Royal Children’s Hospital, Herston Rd, Herston QLD 4029, Australia

**Keywords:** Paediatric dysphagia, Cervical auscultation, Assessment, Oropharyngeal aspiration, Sensitivity, Specificity, Randomised controlled trial

## Abstract

**Background:**

Oropharyngeal aspiration (OPA) can lead to recurrent respiratory illnesses and chronic lung disease in children. Current clinical feeding evaluations performed by speech pathologists have poor reliability in detecting OPA when compared to radiological procedures such as the modified barium swallow (MBS). Improved ability to diagnose OPA accurately via clinical evaluation potentially reduces reliance on expensive, less readily available radiological procedures. Our study investigates the utility of adding cervical auscultation (CA), a technique of listening to swallowing sounds, in improving the diagnostic accuracy of a clinical evaluation for the detection of OPA.

**Methods:**

We plan an open, unblinded, randomised controlled trial at a paediatric tertiary teaching hospital. Two hundred and sixteen children fulfilling the inclusion criteria will be randomised to one of the two clinical assessment techniques for the clinical detection of OPA: (1) clinical feeding evaluation only (CFE) group or (2) clinical feeding evaluation with cervical auscultation (CFE + CA) group. All children will then undergo an MBS to determine radiologically assessed OPA. The primary outcome is the presence or absence of OPA, as determined on MBS using the Penetration-Aspiration Scale. Our main objective is to determine the sensitivity, specificity, negative and positive predictive values of ‘CFE + CA’ versus ‘CFE’ only compared to MBS-identified OPA.

**Discussion:**

Early detection and appropriate management of OPA is important to prevent chronic pulmonary disease and poor growth in children. As the reliability of CFE to detect OPA is low, a technique that can improve the diagnostic accuracy of the CFE will help minimise consequences to the paediatric respiratory system. Cervical auscultation is a technique that has previously been documented as a clinical adjunct to the CFE; however, no published RCTs addressing the reliability of this technique in children exist. Our study will be the first to establish the utility of CA in assessing and diagnosing OPA risk in young children.

**Trial registration:**

Australia and New Zealand Clinical Trials Register (ANZCTR) number ACTRN12613000589785.

## Background

Feeding difficulties in children encompass a wide spectrum including delayed feeding skills development, disordered sensory processing, preferences for a limited range of food types or textures, aversive feeding behaviours resulting in food refusal and/or difficulty with swallowing (oropharyngeal dysphagia) [1]. Oropharyngeal dysphagia is the term used to define dysphagia involving the oral and/or pharyngeal phases of swallowing. Oropharyngeal dysphagia is clinically important in children as its consequences may include inadequate nutritional intake, dehydration and oropharyngeal aspiration (OPA) [2]. OPA is defined as the entry of fluids, food particles and/or oral secretions into the airway below the level of the true vocal cords [3]. OPA is associated with acute respiratory sequelae such as apnea, tachypnea and pneumonia and chronic sequelae such as chronic cough and bronchiectasis [3,4]. Thus, early detection and appropriate management of OPA is important to prevent chronic pulmonary disease and poor growth in children.

Proving the diagnosis of recurrent OPA as the reason for persistent or chronic respiratory systems remains elusive in respiratory medicine [4]. The first step in evaluating a child for oropharyngeal dysphagia is often a clinical feeding examination (CFE) conducted by a speech pathologist. It is subjective [5,6] but inexpensive, non-invasive, time efficient and repeatable. The CFE involves a case history and mealtime observations of the child’s oral sensorimotor, feeding and swallowing skills [5,7-9]. However, the examination only provides an estimation of the co-ordination and movement of food/fluids through the pharyngeal phase. This is determined through visual observation of laryngeal movement [10], palpation of laryngeal movement [11] and listening to clinical features (e.g. cough [12,13], wet voice [5,14], choking, voice change) [12,14]. In particular, clinical features such as wet voice and wet breathing have been significantly associated with OPA detected on MBS [5]. In children, high sensitivity (92%) on fluids but low sensitivity (33%) on solids for the detection of OPA via CFE has been documented in comparison to MBS findings [12]. Similarly, the CFE in adults is limited by low sensitivity and specificity values for the identification of OPA when compared with MBS [9,15,16]. This limitation to the accurate identification and assessment of OPA means many children require further objective assessment, such as a MBS or fiberoptic endoscopic evaluation of swallowing (FEES), to more accurately detect and identify OPA on a variety of food textures and fluid consistencies.

MBS and FEES have comparable sensitivities [17,18] and reliably detects OPA [15,17,19-22], especially when the Penetration-Aspiration Scale (PAS) is used [21,23]. However, both procedures are usually limited to specialist paediatric tertiary centres, are expensive, and require the expertise of a paediatric speech pathologist and radiologist or an otolaryngologist. These may not be readily available because of scheduling requirements of personnel, suites and equipment. Furthermore, MBS involves radiation although at acceptable levels [24].

To improve the diagnostic accuracy of the CFE for detecting OPA, several approaches to the examination have been studied. These include combining the CFE with water swallow challenges [7,25-27], trial swallows using different viscosities [8,13], pulse oximetry [25] and using cervical auscultation (CA). CA is a portable, non-invasive technique that uses a stethoscope to detect cervical sounds generated during the swallow and breath sounds pre- and post-swallow. It is based on the premise that fluid flow through the upper oesophageal sphincter can be heard by listening to the cervical neck region. Higher OPA agreement between CFE + CA (76%), rather than CFE only (42%), when compared to MBS results have been reported in separate adult studies [16,28]. Further, unpublished data titled “Impact of cervical auscultation on accuracy of clinical evaluation in predicting penetration/aspiration in a paediatric population” by Eicher et al. presented at a CA workshop in Virginia (1994) compared CFE (*n* = 15) and CFE + CA (*n* = 41) with MBS findings in 56 children (aged 1–312 months) and found that CFE + CA had a higher agreement with MBS results than CFE only (83% vs. 76%) [29]. CFE + CA had 89% sensitivity and 83% specificity in detecting OPA/penetration compared to MBS. Other adult studies on CA used to accurately identify OPA have described sensitivities of 62-94% and specificities of 66-70% when compared to MBS [30,31]. Current studies documenting the validity of CA in conjunction with the CFE are limited to the adult population [28,30-33] and have methodological limitations such as small subject numbers [30,31,33], observational study designs [28,30-33], clinician bias [31], selection bias with large age differences between comparative groups [30,31], and undefined abnormal swallowing sound parameters [31,32]. As such, there are no randomised controlled trials that have examined the utility of CA in adults and children. Hence, a better understanding of the diagnostic value of this technique for improving OPA detection is required before it can be used routinely in the clinical setting.

### Aims of the study

Our study investigates the utility of cervical auscultation (CA) in the assessment and diagnosis of OPA in children. The primary aim is to determine whether the CFE combined with CA increases the detection of OPA determined by MBS, compared to the CFE only. We hypothesise that the use of CA (compared to not using CA) as an adjunctive clinical tool to assess oropharyngeal dysphagia improves the detection of OPA in children as assessed with the current gold standard, MBS.

Our secondary aim is to determine the perceptual characteristics of common typical respiratory and swallow sound patterns and/or descriptors pre- and post-swallow in children with OPA.

## Methods/Design

### Study design

Open, randomised controlled study. Children randomised to two groups (CFE only vs. CFE + CA) for detection of OPA and compared to that detected on MBS.

### Participants

Inclusion criteria: Children aged < 18 years who are referred for a CFE or MBS at the Royal Children’s Hospital, Brisbane, Australia.

Exclusion criteria: Children deemed medically unfit to complete a CFE and MBS, as determined by the treating medical team. Children can only participate for one series of evaluations.

### Recruitment

The parents/guardians of eligible children will be initially approached by the clinical speech pathologist responsible for the child’s assessment. Parents/guardians interested in participating will then be referred to the principle investigator (TTF). The study will be explained using written information statements and face-to-face interviews. Written informed consent will be obtained from the parents/guardians of children enrolled. Written informed assent will also be obtained from young people aged 12 years and over.

### Randomisation

A computer-generated simple randomisation list, stratified by age (<1 year or ≥1 year), was produced by an external statistician. Allocation concealment will be maintained by group allocation (i.e. CFE or CFE + CA) being concealed in sequentially numbered, opaque envelopes until after consent and baseline information has been obtained. The envelope is opened in front of the treating speech pathologist and caregiver prior to the commencement of their feeding examination.

We stratified by age because swallowing function is influenced by age. The cutoff of 12 months was chosen based on two factors: (1) the swallow-respiration pattern changes at 1 week and between 6 to 12 months of age [34]; (2) anatomical maturity that occurs at approximately 12 months that impacts on a child’s ability to feed more efficiently [35]. These maturity factors include fusion of the mandible halving to improve jaw grading ability, fat pads reducing to increase the oral cavity size and the position of the trachea being more vertical in toddlers than infants.

### Sample size

We plan a sample size of 216 children based on previous research [29] in which the sensitivity of CFE for OPA was 76%. Having 108 children per group provides 80% power (two-sided α = 0.05) to detect a 15% difference between the two groups. A smaller difference between groups was considered insufficient to influence clinical practice. Allowing for 8% attrition (due to unlikely loss to follow-up with both time points at close proximity to each other), a total of 233 children will be enrolled.

### Standardisation of speech pathologists performing CFE + CA

Prior to the commencement of the study, all speech pathologists involved in the assessment of participants will be asked to read through and familiarise themselves with a folder containing a (1) swallowing sound definition sheet; (2) a compact disc (CD) of ten audio examples of normal swallows of puree, lumpy mash, biscuit (chewable) and thin fluids (bottle and cup) in children and one adult; (3) a sheet containing website links to respiratory sound examples of wheeze, stridor, crackles and normal breathing in children; (4) the data collection form. This training package was provided to help attune the clinician’s perception of swallowing sounds and definitions prior to their involvement in the study. The swallowing sounds definition sheet is in Additional file 1.

### Inter-rater reliability

Inter-rater reliability for the detection of OPA will be determined on 20 swallowing sounds scored independently by four speech pathologists who have familarised themselves with the training package, as outlined above. Intra-rater reliability will also be determined for these same 20 sounds. Three swallowing sound clips of swallows from healthy children (with no oropharyngeal aspiration/penetration), four sound clips of abnormal swallows (from patients with radiologically defined oropharyngeal aspiration) and three sound clips of abnormal swallows (from patients with radiologically defined penetration) will be recorded onto a CD twice in random order based on a computer-generated randomisation list, giving a total of 20 examples.

Good to very good intra-rater reliability kappa scores [36] will be accepted for continued involvement in the study. Speech pathologists who do not meet this criterion will receive specific training with the researcher to discuss perceptual characteristics of normal and OPA identified swallows, as confirmed on MBS.

### Procedure and equipment

#### Clinical feeding examination with cervical auscultation (CFE + CA)

Standardised procedures and data collection will be undertaken. Children may be fed by their caregiver, treating speech pathologist or nurse, depending on the child’s preference and/or medical needs.

Swallowing sounds are recorded using an omnidirectional condenser microphone with sensitivity at 1 kHz of 10 mV/Pa, impedance 200 and frequency range 20 to 20,000 Hz (Model C417, AKG Acoustics, Vienna, Austria) [37]. The microphone is attached (by tape) on the lateral border of trachea, immediately inferior to the cricoid cartilage, by the principal researcher only for consistency. Recordings of swallow sounds will be digitally recorded (Digital H4n Handy Recorder, Zoom Corp., Tokyo, Japan). Nasal airflow direction will be measured by an infant- or paediatric-sized cannula placed at the entrance of each nostril and secured firmly behind the ears [34,38]. The quality of sound is monitored consistently throughout the session by listening to extraneous noises (e.g. carotid artery, room noises, reduction of skin-to-skin contact). The researcher or clinician monitors the sound quality and adjustments are made where necessary. To confirm the onset of a swallow, a digital video recorder (model DCR-DVD605E, Sony Corp., Tokyo, Japan) will be used to provide visuals of laryngeal movement associated with the swallow. Nasal airflow data and visuals of the child’s performance will be simultaneously recorded onto the Digital Swallowing Workstation (KayPENTAX, PENTAX Medical Co., NJ, USA). A vocal signal will be used at the beginning of all assessments so synchronisation of acoustic, nasal airflow and visual data can be undertaken. The treating speech pathologist will wear headphones (Model ATH-M50, Audio-Technica, Taiwan) to access the recorded swallowing sounds during the assessment.

Children will be offered three bites or sips/mouthfuls of each age-appropriate food/fluid item (Table 1) followed by free eating/drinking to finish the test meal [39,40].

**Table 1:** **Standard presentation of food and fluids during CFE, CFE** + **CA or MBS**

**Age**	**Food and fluid**
4 – 6 months	● Puree
	● Thin fluids via breast or bottle feeding
8 – 12 months	● Puree
	● Lumpy mash
	● Thin fluids via breast, bottle or spout cup
1 – 18 years	● Puree
	● Lumpy mash
	● Chewable solids (biscuit)
	● Thin fluids via breast, bottle or open cup

Three bites or sips of food or fluid are consistent with normative data obtained previously in adults [41,42] and allow for variations in normal swallowing [32,43]. However, the protocol can be modified at the discretion of the speech pathologist depending on the presentation of the child’s observed feeding and swallowing skills. Reasons for potential protocol modification include:

● Suspected OPA on thin fluids, requiring presentation of thickened fluids prior to thin fluids

● Significantly delayed chewing skills, requiring elimination of an age-appropriate solid consistency

● Reduced compliance from the child, requiring early termination of the session

#### Clinical Feeding Examination (CFE)

The procedure for CFE is similar to the CFE + CA process; however, the treating speech pathologist will not wear the headphones to access the recorded swallowing sounds during the assessment.

#### Modified Barium Swallow (MBS)

After the clinical evaluation (CFE + CA or CFE), all children will undergo an MBS to objectively define the presence or absence of OPA. The MBS will occur within 2 weeks following the CFE, pending clinical constraints.

During the MBS, swallowing sounds and video recording will be undertaken using the same procedure described above. The MBS will be simultaneously recorded onto a Digital Swallowing Workstation (KayPENTAX, PENTAX Medical Co., USA). A vocal signal will occur at the beginning of all procedures for ease of later synchronising of acoustic and exam data. Our standard clinical protocol is adapted from previous research protocol recommendations for paediatric MBS [3,5,24,44,45].

The presence and severity of OPA will be determined using the Penetration-Aspiration Scale (PAS) [23,46,47]. The PAS describes the severity of penetration of radiology contrast into the airway and the airway response to the penetration using an ordinal 1 to 8 scale, where 1 = no aspiration and 8 = silent aspiration. This scale has high inter- and intra-rater reliability for determining the severity rating of OPA [15,21,23]. PAS scores will be determined for each food and fluid consistency trialed in the MBS. A score of 6, 7 or 8 will be classified as OPA present, while scores of 1, 2, 3, 4 and 5 will be classified as OPA absent.

### Data collection

#### Clinical signs of OPA (both groups)

All speech pathologists will adhere to Speech Pathology Australia’s Dysphagia Clinical Guidelines when completing the CFE [48]. CFE parameters of coughing, gagging, voice changes, colour changes, delayed swallow, abnormal laryngeal elevation, oxygen desaturation, eye tearing and wet breathing have been associated with oropharyngeal aspiration when a combination of any of the above are observed during the clinical feeding examination [5,12-14,35,49-53]. In particular, cough is the most significant and consistent clinical sign suggestive of OPA [5,12]. Multiple swallows and laboured breathing have been included as clinical signs suggestive of increased risk of OPA. We have also included: snuffly nose, rattly chest and fremitus as clinical signs of OPA that have been previously observed in clinical practice.

#### Perceptual parameters (CFE + CA group only)

Perceptual parameters for sounds heard throughout the pre-swallow, swallow and post-swallow phases are listed in Table 2.

**Table 2:** **Pre-swallow, swallow and post-swallow sound parameters used in CFE** + **CA**

**Pre-swallow**	**Swallow**	**Post-swallow**
Normal breathing [54]	Crisp and clear ‘distinct’ [56]	Normal breathing [54]
Wet breathing [5,54]	Quick [54]	Wet breathing [5,54]
Rattly chest	Loud	Rattly chest
Grunting	Initial discrete sound (IDS) [32,55,57]	Grunting
Crackles	Bolus transit sound (BTS) [32,55,57]	Crackles
Stridor	Final discrete sound (FDS) [32,57]	Stridor
Wheeze	Glottal release sound (GRS) [55]	Wheeze
Throat clearing	Coordinated	Throat clearing
Coughing [12-14]	Uncoordinated	Coughing [12-14]

Pre- and post-swallow sounds are predominantly respiratory sounds that have been heard in association with aspiration pneumonia. The parameter ‘normal breathing’ is based on Rommel and colleagues’ [54] study of swallowing sounds in children, while ‘wet breathing’ is obtained from Weir and colleagues’ [5] findings of a statistically significant correlation between OPA and the clinical feature of ‘wet breathing’. Coughing and throat clearing were included as cough has been associated with OPA [12-14]. Swallow sounds were based on features previously described in the normal swallows of adults [32,55,56] and infants [54,57].

#### Outcome measures

PAS [23,46,47] scores will be used to objectively determine presence or absence of OPA on MBS, with a score of 6–8 indicating OPA. For CFE, one or more ticked clinical signs of OPA (cough, gagging, voice changes, colour changes, unable to initiate swallow, abnormal laryngeal elevation, oxygen desaturation, eye tearing, multiple swallows, choking, wet breathing, laboured breathing, fremitus, snuffly nose/stertor) [5,12-14,35,49-53] and clinician decision (yes/no) for suspected OPA. For CFE + CA, evaluation was as for CFE outcome measurements and clinician decision (yes/no) for suspected OPA based on pre-, during and post-swallow sound parameters, as listed in Table 2. Presence or absence of OPA is determined by the clinician across all observed swallows for each given texture/consistency in the CFE and MBS. Presence of OPA need only occur once for a child to be marked positive for OPA on that consistency/texture.

### Statistical analyses

Baseline characteristics of the two groups will be presented as means/medians or proportions and their 95% confidence intervals and compared using *t*-tests for normally distributed data, using chi-square tests for proportions and the appropriate non-parametric methods for non-normally distributed data. Baseline demographic and clinical characteristics for those who do and do not withdraw will also be completed. A secondary analysis to re-present the primary results for each of the strata (<1 year and >1 year) will be done to account for the stratification.

For our primary aim, agreement between OPA determined clinical and MBS assessments will be calculated using percent agreement and a kappa coefficient. All evaluable data will be used minus the 8% of dropouts. Taking MBS determination of presence or absence of OPA as the gold standard, sensitivity, specificity, negative predictor value (NPV), positive predictor value (PPV), pretest probability and likelihood ratios will also be calculated. Likelihood ratios will also be calculated.

For our secondary aim, categorical variables of pre-, during and post-swallowing sound parameters will be analysed using a chi-square test to determine which sound qualities are significantly associated with OPA. Multivariate regression analysis will be used to determine whether age, gender, texture, method of intake or clinician level of experience are independent predictors associated with the absence/presence of OPA. All variables will be analysed univariably, then include all those with *p* < 0.15 in a multivariable model. Forward stepwise regression modelling will be employed to identify variables independently associated with OPA and account for interaction effects. The *a priori* statistical level of significance is *p* < 0.05. Missing data points will be coded as a number on the database and analysis completed on the remaining data.

### Ethics approval

Ethics approvals were obtained from the University of Queensland (no.2011001295) and the Children’s Health Services Human Research Ethics Committee (HREC/11/QRCH/52). All families will give written consent to participate and they are able to withdraw their child from the study at any time without explanation or penalty from the research team and staff at the Royal Children’s Hospital.

## Discussion

Assessment of oropharyngeal dysphagia is the most common reason for referral to speech pathologists in tertiary paediatric hospitals. OPA, if left unmanaged, may result in substantial acute and chronic respiratory morbidity [4]. Current diagnostic techniques are limited by accessibility and cost. CA is a tool that potentially improves the clinical assessment of children with oropharyngeal dysphagia at risk of OPA, particularly in regions where MBS is not readily available. This unique study will therefore inform the clinical care and management of children with oropharyngeal dysphagia.

Using CA is dependent on sound differentiation. The link between perceptual and acoustic sound parameters to differentiate between normal and abnormal swallow sounds has been described in three studies that used different parameters [54,57,58]. Rommel et al. [54] collected perceptual and acoustic swallow parameters on 17 healthy children and 9 children with OPA. They defined perceptual swallowing parameters as “abnormal” (flushing sound of liquid heard prior to swallow, coughing or throat clearing after the swallow and/or laryngeal stridor) or “normal” (clear breath sounds heard after the swallow during post-swallow inspiration or expiration) [54]. Statistically significant differences in acoustic parameters of peak amplitude (ranges 16, 25) (*p* < 0.0001) and relative amplitude fundamental frequency (means −11, -16) (*p* < 0.05) data between normal and abnormal perceptual swallow sounds were reported. However, the study had several limitations including a small sample size (*n* = 26), poor matching between groups (children were not age-matched, which is important as age influences swallowing function), and limited data on different viscosities and textures with only thin fluids data reported. Further data on acoustic and perceptual parameters of swallowing on a variety of food and fluid consistencies are required to see if this result is generalisable to other consistencies, apart from thin fluids.

Vice et al. [57] described different sound types in swallowing on a study that evaluated six healthy newborn (within the first 2 days post delivery) infants. They found that the initial discrete sound (IDS) heard at the beginning of the bolus transit sound (BTS) became more uniform with increasing age [57]. Swallow sounds were recorded using an accelerometer and subsequently transferred on to a signal file manager. From these files, waveforms of the swallow sound against time (seconds) and amplitude (volts) were obtained. The IDS was consistently objectively identified from the swallow waveform [57]. Study limitations included a small sample size and unclear definitions. In another study, accelerometer recorded swallow sounds, which were converted to waveforms via a signal file manager, demonstrated waveform shape differences on initial discrete sounds between pre-term infants with and without chronic neonatal lung disease (CNLD) [57,58]. The shape of the waveform or “variance index” in infants with CNLD was significantly different from that in infants without CNLD.

Despite the limitations of the three studies above, they provided proof of concept. Perceptual parameters of swallowing sounds can be linked to objective acoustic data in infants and young children and a differentiation between normal and abnormal swallows is possible perceptually and acoustically. Our study has therefore been designed to build upon previous CA research and addresses the limitations of those studies.

Limitations of this study protocol include variability in clinical assessment given the number of clinicians conducting a clinical feeding examination and delayed scheduling of the MBS following the clinical feeding examination (within a 2-week timeframe). To minimise clinician variability in assessment of swallowing sounds, all clinicians will be required to familiarise themselves with a folder that contains definitions and clip examples of swallow and breath sounds and the data collection booklet. Inter- and intra-rater reliability on the detection of OPA and perceptual swallow sound parameters will also be determined during the course of this study. As with most clinical research studies, scheduling of the MBS post clinical feeding examination is dependent on clinical constraints and staffing, which is difficult to address in a clinical care setting. We acknowledge that using swallowing performance data from one time point to another time point 24 hours to 2 weeks later may have an impact on final results. To reduce this possibility, we are collecting data on time period between clinical feeding examination and MBS. Statistical analysis using interaction models will incorporate this information to determine its potential influence on our findings.

In conclusion, our randomised controlled trial will establish the utility of CA in assessing and diagnosing OPA risk in young children. CA has the potential to identify OPA earlier and minimise the serious consequences on the paediatric respiratory system. If CA improves the diagnostic accuracy of CFE, this will markedly improve the diagnostic abilities of clinicians. It may minimise the need for invasive procedures (e.g., MBS) and tertiary facility assessment of oropharyngeal dysphagia. It may therefore have a substantial impact on both health care costs and service delivery. Particularly in the rural and remote areas, not having to rely on tertiary services will have a positive impact on children and their families in these regions. It is anticipated that this research will influence future clinical practice in the assessment of OPA in children through the inclusion or non-inclusion of CA in routine clinical feeding examinations in children.

## Trial status

Recruitment commenced in October 2012 and will continue for a planned 2-year period.

## Abbreviations

ANZCTR: Australia & New Zealand Clinical Trials Register; BTS: Bolus transit sound; CA: Cervical auscultation; CFE: Clinical feeding evaluation; CNLD: Chronic neonatal lung disease; IDS: Initial discrete sound; MBS: Modified barium swallow; NPV: Negative predictor value; OPA: Oropharyngeal aspiration; PPV: Positive predictor value.

## Competing interests

The authors declare that they have no competing.

## Author’s contributions

TF contributed to the study design and protocol; recruited and collected participant data; and drafted the manuscript. AC and KAW conceived the study and contributed to the study design and protocol. KO contributed to the study design and protocol. KW provided intellectual input for the manuscript. AC, KO and KAW provided critical revision for the manuscript. All authors read and approved the final manuscript.

## Supplementary Material

Additional file 1 **Swallowing sounds definitions sheet.** Three tables with pre-, during and post-swallow sound definitions. Click here for file
