# Supplementary material for: Cervical auscultation in the diagnosis of oropharyngeal aspiration in children: a study protocol for a randomised controlled trial
Source: Trials. 2013 Nov 7;14:377. doi: 10.1186/1745-6215-14-377 (PMC4226202; doi:10.1186/1745-6215-14-377)
Supplement: Additional file 1 — Swallowing sounds definitions sheet. Three tables with pre-, during and post-swallow sound definitions. [file 1745-6215-14-377-S1.doc]

Pre-swallow sounds

|  | Medical definition | Lay definition |
| --- | --- | --- |
| Normal or vesicular breathing | Normal, good, unlabored [respiratory](http://en.wikipedia.org/wiki/Breath) pattern, sometimes known as *quiet breathing* or *resting respiration*  Vesicular breath sounds are soft and low pitched during inspiration and even softer during expiration | When the inspiration and expiration sequence sounds clear |
| Wet breathing | Breathing has a wet sounding quality | Breathing sounds wet |
| Rattly chest | Rhonchal fremitus (bronchial fremitus) is a palpable vibration produced during breathing, caused by partial airway obstruction. | A vibration perceptible on palpation or auscultation or ‘rattly chest’ to touch when hands are placed on the infant’s chest/back. |
| Grunting | A repetitive, low to medium pitch, short, explosive sound which is produced by vocal cord closure during expiration | Vocalisation heard with expiratory breath |
| Crackles - fine | Inspiratory crepitations | Similar to sound of wood burning in a fireplace or the sound of cellophane being crumpled  Brief, discontinuous, popping sounds that are high pitched. More during inspiration. |
| Crackles - coarse | Low in pitch, bubbling sound | Similar to sound when strands of hair are rolled between your fingers near your ear  Discontinuous, brief, popping sounds. Similar to the sound of Velcro being pulled apart. A bubbling sound. More during inspiration. |
| Stridor | Harsh, monophonic, predominantly inspiratory noise. Can be expiratory or biphasic | A harsh, high-pitched, vibratory noise in the throat, particularly when breathing in. Can be when breathing in or out or both |
| Wheeze | Continuous, high-pitched adventitious lung sounds which are superimposed on the normal breath sounds | Flow of air through an airway sounds turbulent |
| Coughing | A cough that sounds barking/brassy, honking, paroxysmal, staccato or wet. | Expeling air from the lungs suddenly with a harsh noise |
| Throat clearing |  | A acute expelling of sound from the throat |

**Swallow sounds**

|  | Description | Listening tips |
| --- | --- | --- |
| Crisp & clear ‘distinct’ | Sounds clear |  |
| Quick | Swallow occurs within 1 s |  |
| Loud | Sound is strongly audible | May vary depending on whether the texture is a fluid, puree or solid. |
| Initial discrete sound (IDS) | Click | Soft , short sound similar to initial sound heard when ears are ‘unblocked.’ |
| Bolus transit sound (BTS) | Food or fluid flushing sound *or*  Flow sound related to turbulences of the fluid | May vary depending on whether the texture is a fluid, puree or solid. Will be more audible on a fluid bolus. |
| Final discrete sound (FDS) | Click | Soft , short sound similar to initial sound heard when ears are ‘unblocked.’  May vary depending on whether the child is taking a single swallow or multiple swallows. Should be more identifiable on single swallows. May occur at the end of a swallow series for continuous drinking or multiple swallow (nor after each swallow) where the airway remains closed until the end of the swallow sequence. |
| Glottal release (GRS) | Short expiration burst of air | May vary depending on whether the child is taking a single swallow or multiple swallows. Should be more identifiable on single swallows. May occur at the end of a swallow series for continuous drinking or multiple swallow (or after each swallow) where the airway remains closed until the end of the swallow sequence. |
| Co-ordinated | Ordered sequence of IDS, BTS +/- FDS and GRS. Sequence occurs within 1s. |  |
| Unco-ordinated | Disordered sequence of IDS, BTS +/- FDS and GRS  *and/or* missing sound component |  |

Post-swallow sounds

|  | Medical definition | Lay definition |
| --- | --- | --- |
| Normal or vesicular breathing | Normal, good, unlabored [respiratory](http://en.wikipedia.org/wiki/Breath) pattern, sometimes known as *quiet breathing* or *resting respiration*  Vesicular breath sounds are soft and low pitched quality during inspiration and even softer during expiration | When the inspiration and expiration sequence sounds clear |
| Wet breathing | Breathing has a wet sounding quality | Breathing sounds wet |
| Rattly chest | Rhonchal fremitus (bronchial fremitus) is a palpable vibration produced during breathing, caused by partial airway obstruction. | A vibration perceptible on palpation or auscultation or ‘rattly chest’ to touch when hands are placed on the infant’s chest/back. |
| Grunting | A repetitive, low to medium pitch, short, explosive sound which is produced by vocal cord closure during expiration | Vocalisation heard with expiratory breath |
| Crackles - fine | Inspiratory crepitations | Similar to sound of wood burning in a fireplace or the sound of cellophane being crumpled |
| Crackles - coarse | Low in pitch, bubbling sound | Similar to sound when strands of hair are rolled between your fingers near your ear |
| Stridor | Harsh, monophonic, predominantly inspiratory noise. Can be expiratory or biphasic | A harsh, high-pitched, vibratory noise in the throat, particularly when breathing in. Can be when breathing in or out or both |
| Wheeze | Continuous, high-pitched adventitious lung sounds which are superimposed on the normal breath sounds | Flow of air through an airway sounds turbulent |
| Coughing | A cough that sounds barking/brassy, honking, paroxysmal, staccato or wet. | Expeling air from the lungs suddenly with a harsh noise |
| Throat clearing |  | A acute expelling of sound from the throat |

**References**
